# A cautionary tale for machine learning generated configurations in presence of a conserved quantity

**DOI:** 10.1038/s41598-021-85683-8

**Published:** 2021-03-18

**Authors:** Ahmadreza Azizi, Michel Pleimling

**Affiliations:** 1 Department of Physics, Virginia Tech, Blacksburg, VA 24061-0435 USA; 2 Center for Soft Matter and Biological Physics, Virginia Tech, Blacksburg, VA 24061-0435 USA; 3 Academy of Integrated Science, Virginia Tech, Blacksburg, VA 24061-0563 USA

**Keywords:** Physics, Condensed-matter physics, Phase transitions and critical phenomena

## Abstract

We investigate the performance of machine learning algorithms trained exclusively with configurations obtained from importance sampling Monte Carlo simulations of the two-dimensional Ising model with conserved magnetization. For supervised machine learning, we use convolutional neural networks and find that the corresponding output not only allows to locate the phase transition point with high precision, it also displays a finite-size scaling characterized by an Ising critical exponent. For unsupervised learning, restricted Boltzmann machines (RBM) are trained to generate new configurations that are then used to compute various quantities. We find that RBM generates configurations with magnetizations and energies forbidden in the original physical system. The RBM generated configurations result in energy density probability distributions with incorrect weights as well as in wrong spatial correlations. We show that shortcomings are also encountered when training RBM with configurations obtained from the non-conserved Ising model.

## Introduction

In recent years machine learning applications have seen a rapid proliferation in almost all fields of science. In condensed matter physics machine learning models, that aim at learning the probability distribution that the input data have been sampled from, have been used to investigate phase transitions^1–19^, characterize quantum circuits^20–23^, predict crystal structures^24–26^, and learn renormalization group flow^27,28^, to name but of few areas of applications.

Machine learning methods, which encompass methods as diverse as principal component analysis^1–5^, support vector machines^6,7^, and variational autoencoders^2,8^, in addition to various neural network architectures^7,12–19^, have been applied to study various properties of physical systems. These models can be grouped into the two broad categories of supervised and unsupervised learning. Supervised learning methods have successfully identified phases of matter and located phase transition points^12,15,16^. In many of these approaches the algorithms are trained over physical configurations, often obtained from importance sampling Monte Carlo simulations, after which they are used to predict the class of a test configuration. For a system with an order-disorder transition, configurations are typically classified as ordered or as disordered. The output from these classifiers, that display a behavior equivalent to an order parameter^12^, can reliably determine the location of phase transition points. For systems with no clear order parameter, machine learning output has been shown to play a role similar to an order parameter which can then be exploited to locate a phase transition^1–4,15,17^. For example, in^1^ principle component analysis was used to extract from spin configurations of the conserved-magnetization Ising model the structure factor which allowed to locate the critical point.

The physics community has also paid much attention to generative learning models, a subset of unsupervised learning methods, that reconstruct configurations for classical^2,9,12^ or quantum^29–32^ systems. The idea behind generative models is to learn the hidden probability distribution underlying the unlabeled input data. Generative adversarial networks^33^ and variational autoencoders^34^ are two types of generative models used for classical systems^2,35,36^. However, more broadly used are restricted Boltzmann machines (RBM)^9,10,37–44^.

Machine learning studies of classical spin systems have almost exclusively focused on non-conserved models. However, conserved quantities can have a major impact on the physical properties of a system as they put strong constraints on the accessible parts of configuration space. In^1^ and^6^ learning algorithms, based either on principal component analysis (PCA) or support vector machines (SVM), were applied on configurations obtained from Monte Carlo (MC) simulations of the two-dimensional conserved-magnetization Ising model. It was shown that for zero magnetization configurations (i.e. configurations with exactly the same number of up and down spins) the output of the algorithms behaved like an order parameter which then allowed to locate the phase transition point.

In the following we report the results of discriminative and generative machine learning on training configurations obtained from Monte Carlo simulations of the Ising model with Kawasaki dynamics and constant magnetization^45^. As binary classifier we use a convolutional neural network (CNN)^46^ and show that the CNN output not only behaves like an order parameter and allows to locate with high precision the critical point separating the ordered and disordered phases, it also displays a finite-size scaling governed by the critical exponent $\nu$. The outputs of PCA and SVM from datasets obtained for the constant-magnetization Ising model do not exhibit the same scaling property. For the generative machine learning we use a restricted Boltzmann machine, trained on datasets obtained from the Ising model with constant (not necessarily zero) magnetization. While RBM allows to obtain good estimates for the average energy, a closer look reveals considerable shortcomings with the RBM generated configurations. It is a not trivial task for RBM to identify a conserved quantity, which, in our case, results in the generation of configurations with magnetization and energy values forbidden in the conserved-magnetization Ising model. We also observe systematic differences between RBM generated configurations and MC generated configurations in the weights of the energy density probability distribution function. Finally, spatial correlations computed using the RBM configurations deviate systematically from spatial configurations obtained in Monte Carlo simulations with spin exchanges. We also revisit machine learning for the non-conserved Ising model and find the same issues with the energy density probability distribution and with the spatial correlations.

## Two-dimensional Ising model with conserved magnetization

As a simple model with a conserved quantity we consider in this work the Ising model with conserved magnetization. Every point with coordinates (*i*, *j*), $i,j=1, \ldots , L$, on a square lattice of linear length *L* is characterized by a classical variable $S_{i,j}$ that can take on only the two values 1 and $-1$. Setting the coupling constant equal to 1, the energy of an arrangement of these spin variables is given by

1 $$\begin{aligned} {{\mathscr {H}}} = - \sum _{i,j=1}^L \left( S_{i,j} S_{i+1,j} + S_{i,j} S_{i,j+1} \right) \end{aligned}$$

with the periodic boundary conditions $S_{L+1,j} = S_{1,j}$ and $S_{i,L+1}=S_{i,1}$. The magnetization density is

2 $$\begin{aligned} M = \frac{1}{L^2} \sum _{i,j=1}^L S_{i,j} ~, \end{aligned}$$

whereas the energy density is

3 $$\begin{aligned} E = \frac{1}{L^2} \sum _{i,j=1}^L S_{i,j} \left( S_{i+1,j} + S_{i,j+1} \right) ~. \end{aligned}$$

In the thermodynamic limit and without additional constraints the two-dimensional Ising model undergoes at the critical temperature (setting $k_B=1$) $T_c=2/\ln (1+ \sqrt{2})$ a continuous phase transition separating the ordered, magnetized phase with non-vanishing magnetization from the disordered phase with zero magnetization.

Fixing the magnetization at some value $M_0$ narrows the space of possible configurations. For $M=M_0=0$ only configurations with exactly 50% of the spins taking on each of the two possible values are accessible. Obviously, the magnetization then does not display anymore the typical behavior of an order parameter when crossing the critical temperature.

We generate for a fixed magnetization density $M = M_0$ independent configurations at a temperature *T* through standard importance sampling Monte Carlo simulations with spin exchange. These configurations are then used for two purposes: (1) to train the machine learning algorithms and (2) to compute energy density probability distributions as well as thermal averages of the energy density $\varepsilon = \langle E \rangle$ (here and in the following $\langle \cdots \rangle$ indicates an average over configurations), the absolute value of the magnetization density

4 $$\begin{aligned} | M | = \left\langle \frac{1}{L^2} | \sum _{i,j=1}^L S_{i,j} | \right\rangle , \end{aligned}$$

and the space-dependent correlations

5 $$\begin{aligned} C(r) = \left\langle \frac{1}{2 L^2} \sum _{i,j=1}^L S_{i,j} \left( S_{i+r,j} + S_{i,j+r} \right) \right\rangle \end{aligned}$$

and compare the values of these quantities with those obtained from the configurations created through machine learning.

**Figure 1:**
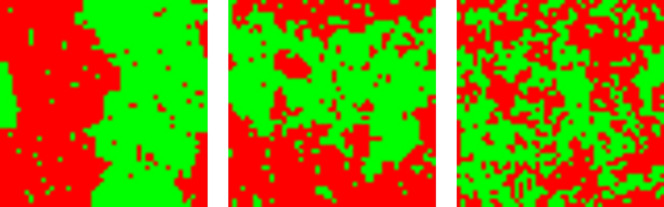
Typical configurations for a system with $L=50$, $M_0=0$ and (from left to right) $T=2$, $T=T_c$, and $T=3$.

Typical configurations with zero magnetization are shown in Fig. 1. Below $T_c$ the system phase separates into two parts with different majority spins. The configuration with the lowest energy, which is the dominating configuration at very low temperatures, is the one where the system is separated into two perfectly ordered halves, with straight interfaces separating the two parts. Increasing the temperature, the interfaces roughen and the halves are less well ordered, see the configuration at $T=2$. Above $T_c$ the large compact regions are broken up and complicated, interlocked clusters remain.

## Convolutional neural network: phase transition with conserved order parameter

In classical, solid states, and quantum physics, machine learning, both supervised and unsupervised, has quickly become a much used tool for the study of phase transitions, as witnessed by recent investigations of spin systems using techniques as diverse as principal component analysis^1–5^, support vector machines^6,7^, variational autoencoders^2,8^, Boltzmann machines^9–11^, fully connected neural networks^12–16^ as well as convolutional neural networks^7,12,15,17–19^. The vast majority of these studies focused on systems without conserved quantities. Only two studies, one using principal component analysis (PCA)^1^, the other support vector machines (SVM)^6^, briefly discussed the two-dimensional Ising model with conserved order parameter. Our goal in the following is to use a convolutional neural network (CNN) based classifier and investigate the phase transition in the two-dimensional Ising model using exclusively configurations with zero magnetization. As we will show, for the two-dimensional Ising model with zero magnetization the output of the CNN displays a critical finite-size scaling behavior not seen when using PCA or SVM.

Our model is a binary classifier with convolutional layers that determines whether a test configuration is in the ordered phase or not. CNNs, which are considered to be the most successful models in image processing problems^46^, are able to extract important features of images, i.e, they learn boundaries of different objects in a given image and classify them accordingly. This is an important property when learning the phase transition in systems with conserved order parameter. Indeed, for $M_0=0$ there is no majority spin state, and configurations are composed of similar clusters for all spin states. Therefore, unlike fully connected neural network models that merely use the number of majority spins in configurations to learn the different thermodynamic phases and therefore fail in cases with conserved magnetization^12^, convolutional neural layers are well suited for the system at hand as they learn the differences between the shapes of spin clusters in the different (ordered and disordered) phases.

**Figure 2:**
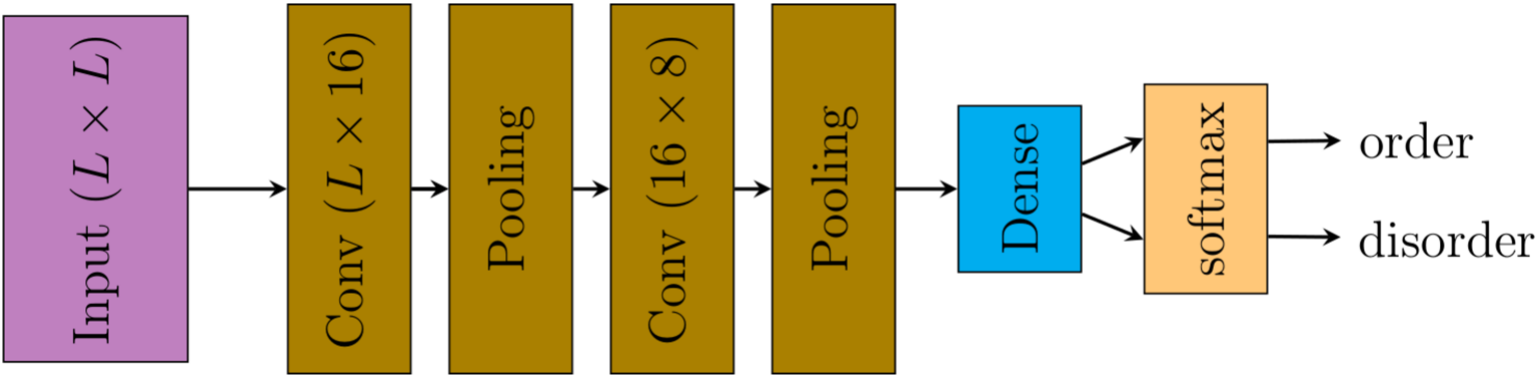
Structure of the convolutional neural network used to investigate the phase transition in the two-dimensional Ising model with conserved magnetization. *L* is the linear dimension of the square lattice.

Figure 2 depicts the structure of our CNN model. It includes two sets of convolutional layer with pooling layer that are followed by a dense layer with a softmax activation function. Therefore the CNN model output is a real number in the range [0, 1] which is the probability of the given configuration being in the ordered phase (of course, as the sum of the probabilities to be in the ordered or disordered phase is one, the output also provides us with the probability that the given configuration is in the disordered phase).

Following the standard protocol for supervised learning of phases, we generate a large number of configurations from importance sampling Monte Carlo simulations of square Ising systems of linear dimension *L* with zero magnetization and Kawasaki spin exchange dynamics. For every system size $L=20, 30, 40, 50$ we generate $10^5$ configurations at temperatures $T=1.0+(n-1) \Delta T$ with $\Delta T = 0.1$ and $n=1, \ldots , 23$. These configurations are then split into training sets (70% of configurations generated at each of the 23 temperatures), validation sets (10% of generated configurations) and test sets (20% of generated configurations). For the training, configurations generated at temperatures $T < T_c$ receive the label ‘1’, whereas configurations generated at $T > T_c$ are labeled as ‘0’. Convergence of the training process, during which the learning algorithm is optimized for a number of epochs using the entire training set, is evaluated by the validation dataset. After each training epoch the model accuracy is measured and the training process is stopped when for three consecutive epochs the model accuracy on the validation dataset is not improved. Finally, the test dataset is used to evaluate the model’s performance.

**Figure 3:**
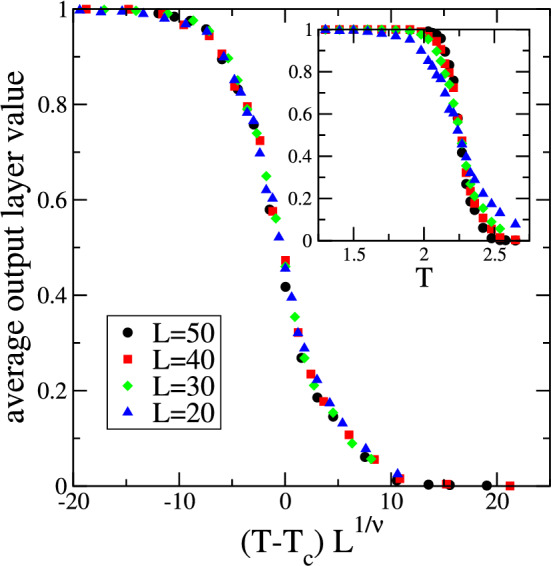
Average output layer value over the test configurations for different system sizes as function of temperature. As shown in the inset, this quantity displays the expected behavior of an order parameter close to a phase transition. Zooming in into the temperature region close to $T_c \approx 2.269$ reveals that the data for all the different system sizes reach the value 0.5 in very close vicinity to $T_c$. As shown in the main figure the average output layer value displays a finite-size scaling behavior governed by the Ising exponent $\nu = 1$. Error bars are comparable to the size of the symbols.

Figure 3 summarizes our results for the average output layer value over the test configurations. As shown in the inset, this quantity, similarly to the outputs obtained from PCA^1^ and SVM^6^, displays the generic behavior of the order parameter in a system with an order-disorder phase transition, with a value close to 1 at low temperatures (ordered phase) and a value close to zero at high temperatures (disordered phase). The deviations from the value 1 below $T_c$ and from the value 0 above $T_c$ are the results of false classifications. These misclassifications have a physical origin and reflect the increase of fluctuations and of diversity of configurations close to a critical point. For the different system sizes, this quantity takes on the value 0.5 in the temperature interval $\left[ 2.25, 2.27 \right]$, in very close proximity to the known critical temperature $T_c = 2/\ln (1+ \sqrt{2}) \approx 2.269$. The average output layer value displays a critical finite-size scaling behavior, see the main panel in Fig. 3, as it is a function of $\left( T - T_c \right) L^{1/\nu }$ with the Ising exponent $\nu =1$. This critical finite-size scaling, which has not been observed in the earlier investigations of the conserved-order-parameter Ising model using machine learning techniques^1,6^, is similar to that encountered in supervised learning of non-conserved spin systems close to their critical point^12,16^.

## Restricted Boltzmann machine: space-dependent correlations

Generative learning, with the goal to capture the probability distribution function underlying the input data and produce new data similar to the input data, is a demanding task. In order to perform this task Torlai and Melko^9^ have used a restricted Boltzmann machine (RBM), a stochastic neural network^47,48^, and have shown that the spin configurations generated in that way yield for the non-conserved Ising model reasonable values for quantities like the average magnetization and energy densities. Subsequently RBMs have been used successfully on other classical^11^ and, especially, quantum systems^29,49,50^. Similar generative approaches have also been used for neural network renormalization group studies^27,28^, as well as in proposals to exploit machine learning for accelerating Monte Carlo simulations^37,51^.

What has been missing in earlier studies using RBMs for the investigation of classical spin systems is a stringent test of the quality of the probability distribution underlying the process of creating representative configurations that are then used to compute (thermal) averages. As already mentioned, RBM generated configurations yield averages for magnetization density and energy density that agree well with those obtained from importance sampling Monte Carlo simulations^9^, but then even rather dissimilar probability distributions can result for some quantities in averages that roughly agree. In the following we show results that point to major differences between RBM generated configurations and configurations obtained from Monte Carlo simulations and used to train the RBM. Most revealing will be the comparison of the energy density probability distributions as well as of spatial correlations (5). Some of these differences come from the fact that RBM can not cope in good ways with conserved quantities. However, we will show via the spatial correlations in the non-conserved Ising model that the observed deficiencies are more general and are not restricted to systems that exhibit a conserved quantity.

### Some technical details

**Figure 4:**
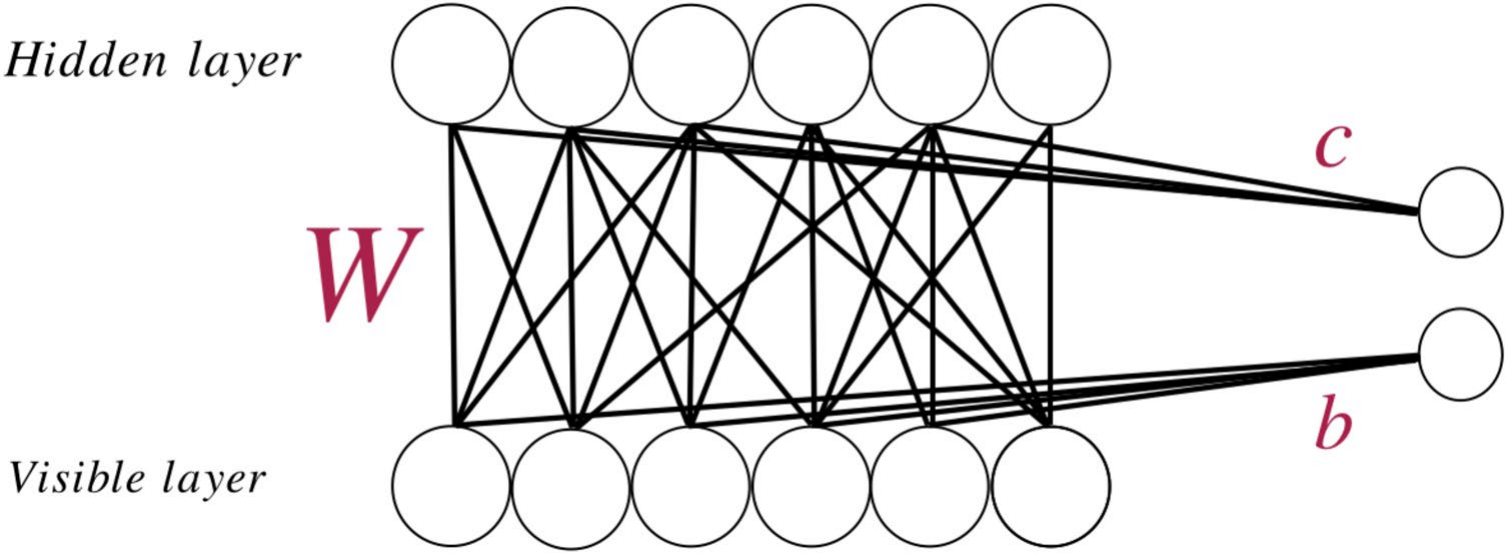
Sample architecture of RBM. In our implementation the number of nodes in the hidden layer is chosen to be identical to the number of nodes in the visible layer. The two nodes on the right hand side represent the bias terms in Eq. (7).

An earlier thorough investigation has shown that a shallow RBM is more efficient to learn the physical properties of Ising models than a deep generalization^10^. Taking this into account, we apply only one hidden layer in our RBM architecture, i.e. we have only two layers, the visible layer *V* and one hidden layer *H*, see Fig. 4. RBMs have been discussed in detail elsewhere^9–11,47,48^. We give in the following only a brief description, with the main intend to provide values for the parameters used in our implementation.

Denoting the nodes in the input layer as $V=\left\{ v_1 , \ldots , v_n \right\}$ and the nodes in the hidden layer as $H = \left\{ h_1 , \ldots , h_m \right\}$, with the hidden nodes only taking on binary values 0 and 1, the joint probability distribution is defined as

6 $$\begin{aligned} P(V,H) = \frac{1}{Z} e^{-E(V,H)} \end{aligned}$$

with the “energy”

7 $$\begin{aligned} E(V,H) = - V^T b - c^T H - V^T W H \end{aligned}$$

and the “partition function” $Z = \sum _{V,H} e^{-E(V,H)}$. Here, *b*, *c*, and *W* contain the model parameters that are trainable through the optimization scheme. It was shown in^9^ and^10^ that for Ising systems $m=n=L^2$ is an appropriate choice for the number of hidden nodes, and we will present in the following results with this number of hidden nodes. We also explored cases with $m > n$ (with up to 200 hidden neurons), but this did not yield substantial improvements.

Summarizing the model parameters as $\theta = \left\{ b, c, W \right\}$ and performing the summation of the hidden layer nodes that take on only the values 0 and 1 yields the likelihood function

8 $$\begin{aligned} P(V | \theta ) = \frac{1}{Z} e^{V^T b} \prod _{j=1}^n \left( 1 + e^{c_j + V^T w_j} \right) \end{aligned}$$

where $w_j$ is the *j*th column in *W*. As optimization method we apply a gradient descent based scheme to update the parameters $\theta$ at each iteration:

9 $$\begin{aligned} \theta _{t+1} - \theta _t = \alpha ~ \nabla \log P(V,\theta ) \end{aligned}$$

with the learning rate $\alpha$. The gradient operator acts on all vector and matrix elements in $\theta = \left\{ b, c, W \right\}$. More specifically, we use the learning rate $\alpha = 5 \times 10^{-3}$, the ‘Adam’ optimization scheme^52^, and the Contrastive Divergence algorithm CD-k with $k=10$ steps^53^. Typically 10,000 spin configurations obtained from Monte Carlo simulations of the Ising model are used for the training.

Once the training phase is done and the optimized values $b^*$, $c^*$, and $W^*$ are found for the parameters, Gibbs sampling is used to generate new configurations. Like CD-k, Gibbs sampling performs a Markov chain between the visible and invisible layers, but this time the chain starts from a random initial configuration in the visible layer. The values $H = \left\{ h_1, \ldots , h_n \right\}$ of the nodes in the invisible layer are computed from $P(H | \theta ^*, V)$. In a second step the values of the visible layer are updated with the help of the probability distribution $P(V | \theta ^*, H)$: the probability that the node *j* in the visible layer takes on the value 1 is

10 $$\begin{aligned} P(v_j =1 | \theta ^*, H) = \frac{\exp \left( b_j^* + h_i w_{ij}^* \right) }{1+ \exp \left( b_j^* + h_i w_{ij}^* \right) }~, \end{aligned}$$

where $h_i$ is the value of node *i* in the hidden layer. After drawing a random number *p* from the interval $\left( 0, 1 \right)$, the node *j* in the visible layer is updated to the value $v_j=1$ when $p \le P(v_j =1 | \theta ^*, H)$ and to the value $v_j=-1$ otherwise. This is repeated *k* times until convergence is reached. We investigated a rather wide range of number of steps *k*, ranging from 2 to 50, and found that in most cases $k=10$ is enough to reach the final converged state. It is worth mentioning that increasing the number of training configurations to 20,000 did not significantly improve the quality of the RBM configurations.

### Ising systems with conserved magnetization

We start by discussing our results for the Ising model with conserved magnetization, which has been the main subject of our investigation. This is followed by a brief discussion of the spatial correlations in the non-conserved Ising model, which illustrates that the observed deficiencies of RBM generated configurations persist in absence of conserved quantities.

In this subsection we use Monte Carlo (MC) generated configurations with conserved magnetization $M_0$ to train the RBM and compare the properties of RBM generated configurations with those obtained from MC. We present results for both $M_0 = 0$ and $M_0 > 0$. The demanding task of generating configurations via RBM only allows to investigate rather small system sizes. The results presented in the following have been obtained with $L=8$ and $L=12$. As already discussed, we applied the CD-K method in order to decrease the convergence time. Since in our experiments the log-likelihood function is not tractable, we monitored the mean absolute value of the trainable parameters and let the training phase continue until this quantity fluctuate around its final value. In this way we check that the trainable variables do not change significantly through the optimization process. Given the above criteria, it usually takes about 1000 epochs on average for our model to converge at the different temperatures.

**Figure 5:**
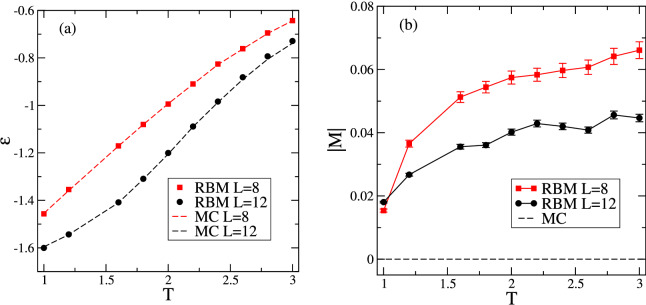
Comparison of averages obtained from RBM generated configurations (symbols) and from configurations generated in importance sampling Monte Carlo (MC) simulations of the conserved-magnetization Ising model with $M_0=0$ (dashed lines). The RBM machine has been trained using the MC configurations. Data for two different system sizes are displayed. Shown are (**a**) the energy density and (**b**) the magnetization density.

Figure 5 compares the RBM and MC averages for the commonly investigated energy and magnetization densities as a function of temperature *T*. For the energy density, see Fig. 5a, we find the same good agreement between RBM and MC averages as observed for the non-conserved case in Ref.^9^. Whereas the average energy density does not hint at any major issues with the RBM generated configurations, this is different for the average absolute value of the magnetization density. As shown in Fig. 5b, the constraint, that the magnetization is $M_0=0$ in all MC configurations used to train RBM, is not captured by the machine learning algorithm, and, consequently, in many RBM generated configurations $M \ne 0$ as there is an excess of one of the spin types. This yields an average absolute value of the magnetization that differs from that of the training configurations. The shortcoming of RBM to identify and reproduce a conserved quantity is also expected to be encountered in other systems as well as for other conserved quantities.

**Figure 6:**
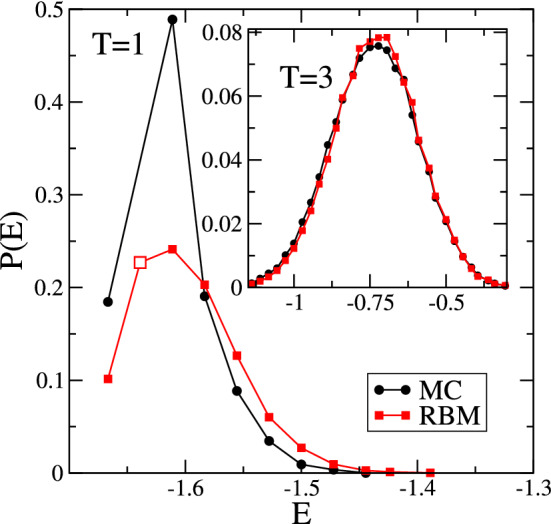
Probability distributions for the energy density for RBM and MC with $L=12$. The main image compares the probability distributions at $T=1$, i.e. below the critical temperature, whereas the inset shows the probability distribution at $T=3$, above the critical temperature. The white square indicates an energy value that can not be accessed in MC simulations of the conserved-magnetization Ising model with $M_0=0$, but is present in almost 23% of the the RBM generated configurations. For $T=1$ we used 10,000 configurations to produce the probability distribution, whereas 100,000 generated configurations were used for $T=3$.

The probability distribution for the energy density, displayed in Fig. 6, shows marked differences between RBM and MC, and this even though the average energy densities closely agree, see Fig. 5a. Below $T_c$, and as exemplified by the data for $T=1$ in Fig. 6, RBM fails to learn the correct weights for the different energy levels. Furthermore, RBM generates configurations with spin arrangements that break the conserved quantity and, in some cases, yield configurations with energies that are not accessible in the Ising model with conserved order parameter. An example is provided in the figure by the large white rectangle. Differences in weights can also be seen for $T > T_c$, see the inset for an example at $T=3$, especially in the increasing part at low energies and around the peak maximum. We checked that these differences do not change substantially when doubling the number of configurations used to train the RBM.

**Figure 7:**
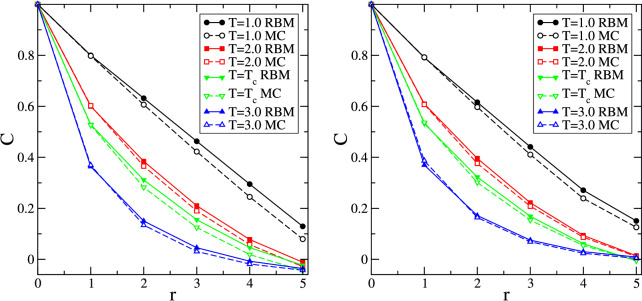
(Left) Comparison of the average space-dependent correlations *C*(*r*) obtained from MC configurations with $M_0=0$ with corresponding RBM configurations that are generated after training over the same MC configurations. Data for four different temperatures are shown for a system with $L=12$. Error bars are smaller than the symbol sizes. (Right) The same, but now MC configurations with an excess of 30 spins of one of the spin types are used.

As the space-dependent correlation function *C*(*r*) is proportional to the energy density for $r=1$ (indeed $C(r=1)=- \frac{1}{2} \varepsilon$, see Eqs. (3) and (5)), we have for $C(r=1)$ the same agreement between MC and RBM as we have for the average energy density. However, as shown in Fig. 7, marked differences show up for $r \ge 2$. These deviations between the spatial correlations computed from RBM and MC configurations point to challenges encountered by RBM when extracting information from the training configuration that go beyond the simple nearest neighbor correlations. These deviations, which are largest for temperatures $T \le T_c$, are decreasing for larger temperatures and are expected to vanish completely for very high temperatures where the typical configuration is a highly disordered one with only very small connected clusters and very short-ranged correlations.

The struggle of RBM to create a reasonable set of configurations for a fixed value of the magnetization is not restricted to the special case $M_0=0$, where the same number of spins is encountered for each spin type, but it also persists for other values of $M_0$. For this we run Monte Carlo simulations with spin exchanges for systems with fixed, but different, numbers of spins for both types, so that the magnetization is fixed at some value $M_0 > 0$. The configurations obtained from these simulations are then used to compute the MC quantities and to train a RBM in order to generate new configurations. These newly generated configurations are then used to compute RBM quantities. In Fig. 7 we show as an example the difference in spatial correlations for configurations with $L=12$ when in the MC dataset the number of majority spins exceeds by 30 the number of minority spins, yielding the constant value $M_0 = 0.2083$ for the magnetization. Systematic differences between RBM and MC are also observed for the other quantities investigated in this study. RBM fares slightly better when increasing the value of the fixed magnetization $M_0$ as it is easier for RBM to learn the energy distribution due to the fact that the number of accessible energies changes with $M_0$, going from the largest number for $M_0=0$ to a single accessible energy for $M_0=1$.

### Ising systems with non-conserved magnetization

As the earlier accounts highlighting successes of RBM in creating configurations of classical systems^9,11^ were dealing with systems without conserved quantities, we found it of interest to have a fresh look at the Ising model without conserved magnetization, following closely what has been done previously, but to compute other quantities than merely the average magnetization and energy. We did check, though, that our results for the magnetization and energy densities agree with those obtained in^9^.

**Figure 8:**
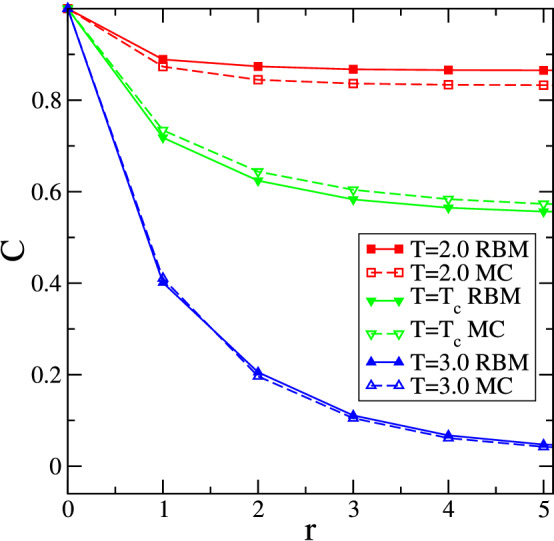
Comparison of the average space-dependent correlations *C*(*r*) obtained from configurations generated in Monte Carlo simulations of the non-conserved Ising model as well as from RBM configurations generated after training with the same MC configurations. Data at different temperatures are shown for a system with $L=12$. Error bars are smaller than the symbol sizes.

As shown in Fig. 8, even when trained using configurations without conserved magnetization, RBM fails to correctly capture longer range correlations at temperatures $T \le T_c$ (similar differences can also be seen in the figures of reference^44^). Similarly, deviations between RBM and MC are observed in the energy density probability distributions (not shown). These are sobering observations, as they mean that RBM generated configurations generically do not reproduce important physical aspects of the system used to train the machine (see^54^ for a related discussion of shortcomings in the joint magnetization and energy probability distribution in small Ising systems computed from RBM generated configurations). The fact that RBM generated configurations do not have the same statistical properties as the configurations obtained from importance sampling Monte Carlo simulations makes it doubtful that machine learning generated configurations can be used safely for speeding up numerical simulations, as proposed in^37,51^.

## Conclusion

Recent years have seen a strong increase of physicists’ interest in supervised and unsupervised machine learning. Some of the most promising applications are found in quantum physics^29–32^ and in materials discovery^24–26^. Classical systems have mainly been used as test cases in order to gain a better understanding of the power of machine learning algorithms by comparing their outputs with well understood results from statistical physics.

Previous studies of standard interacting spin systems have revealed that machine learning algorithms can identify different phases, locate phase transition points, determine the order of a phase transition, and generate configurations that yield average values for magnetization and energy densities in agreement with values obtained from importance sampling Monte Carlo simulations. While these are remarkable achievements, they are often qualitative or are dealing with a few selected averaged quantities that do not fully characterize the system.

The vast majority of machine learning studies of classical spin systems considered situations without conserved quantities. However, conserved quantities restrict the accessible part of configuration space which often results for finite systems in modified physical properties. Two studies briefly discussed the two-dimensional Ising model with conserved (zero) magnetization and showed that standard machine learning techniques (principal component analysis^1^ and support vector machine^6^) allow to locate the phase transition point using the machine learning output. No past attempts were made to use machine learning for more demanding tasks than the transition point location in systems with a conserved quantity.

In this paper we have presented two different types of results for the two-dimensional Ising model with conserved magnetization. In the first part of the paper we have shown that supervised machine learning using convolutional neural networks not only allows to locate the phase transition temperature with high precision, it also yields as output a quantity that behaves like an order parameter and displays a critical finite-size scaling governed by the exponent of the two-dimensional Ising model. In the previous studies of the conserved-magnetization Ising model the outputs from PCA and SVM merely allowed to locate the phase transition point without permitting to determine the value of a critical exponent through finite-size scaling. The second part of our paper has revealed physically relevant shortcomings of the configurations generated from a restricted Boltzmann machine trained with configurations obtained from the conserved-magnetization Ising model. In the optimization process, not only is RBM not penalized when generating configurations with magnetizations that differ from that of the training dataset, it also yields changes in weights for allowed energies as well as space-dependent correlations that differ from those obtained in importance sampling Monte Carlo simulations of the original system. These observations have triggered us to also revisit the non-conserved Ising model: whereas RBM yields for that system an acceptable agreement with Monte Carlo simulations for quantities like the average magnetization and the average energy^9^, a closer look reveals that RBM does not yield very good approximations for the energy density probability distributions nor does it produce space-dependent correlations that agree with those obtained from Monte Carlo simulations. Therefore, the statistical properties of configurations generated by RBM present marked differences from those used in the training dataset, and this independently whether or not a conserved quantity is present.

Our results for the RBM configurations beg the question whether RBM generated configurations should be used at all. This will depend on the application and on whether rough estimates of average quantities are needed or whether high precision data are required that fulfill the statistical properties of the original physical system. Especially worrisome seem to be the proposals to use machine learning generated configurations to speed up numerical simulations^37,51^, as this approach may inject into the Markov chain configurations with statistical properties that differ from those of the original system. It will depend on the system and on the physical question at hand whether this is acceptable and allows to obtain results that are physically meaningful for the original system.

Our investigation exclusively dealt with classical spin systems, however similar issues with statistical properties can also be expected to show up in some quantum applications. We hope that our work will trigger more rigorous approaches to use machine learning outputs in the different fields of physics.
